# Persistence of Antibody Responses to the SARS-CoV-2 in Dialysis Patients and Renal Transplant Recipients Recovered from COVID-19

**DOI:** 10.3390/pathogens10101289

**Published:** 2021-10-06

**Authors:** Maria Cappuccilli, Paolo Ferdinando Bruno, Alessandra Spazzoli, Matteo Righini, Marta Flachi, Simona Semprini, Laura Grumiro, Maria Michela Marino, Pasqua Schiavone, Elisabetta Fabbri, Michela Fantini, Andrea Buscaroli, Angelo Rigotti, Gaetano La Manna, Vittorio Sambri, Giovanni Mosconi

**Affiliations:** 1 Nephrology, Dialysis and Renal Transplant Unit, IRCCS-Azienda Ospedaliero-Universitaria di Bologna, Alma Mater Studiorum University of Bologna, 40138 Bologna, Italy; maria.cappuccilli@unibo.it (M.C.); gaetano.lamanna@unibo.it (G.L.M.); 2 Nephrology and Dialysis Unit, AUSL Romagna Morgagni-Pierantoni Hospital, 47121 Forlì, Italy; paoloferdinando.bruno@auslromagna.it (P.F.B.); alessandra.spazzoli@auslromagna.it (A.S.); 3 Nephrology and Dialysis Unit, AUSL Romagna S. Maria delle Croci Hospital, 48121 Ravenna, Italy; matteo.righini3@studio.unibo.it (M.R.); andrea.buscaroli@auslromagna.it (A.B.); 4 Nephrology and Dialysis Unit, AUSL Romagna Infermi Hospital, 47923 Rimini, Italy; marta.flachi@auslromagna.it (M.F.); angelo.rigotti@auslromagna.it (A.R.); 5 Unit of Microbiology, AUSL Romagna Laboratory, 47023 Pievesestina, Italy; simona.semprini@auslromagna.it (S.S.); laura.grumiro@auslromagna.it (L.G.); mariamichela.marino@auslromagna.it (M.M.M.); pasqua.schiavone@auslromagna.it (P.S.); vittorio.sambri@auslromagna.it (V.S.); 6 Local Healthcare Authority of Romagna (AUSL Romagna), 48121 Ravenna, Italy; elisabetta.fabbri2@auslromagna.it (E.F.); michela.fantini@auslromagna.it (M.F.)

**Keywords:** antibody persistence, COVID-19, humoral immune response, immunodepressed patients, renal transplant recipients, neutralizing antibodies, SARS-CoV-2 S1/S2

## Abstract

Nephropathic subjects with impaired immune responses show dramatically high infection rates of coronavirus disease 2019 (COVID-19). This work evaluated the ability to acquire and maintain protective antibodies over time in 26 hemodialysis patients and 21 kidney transplant recipients. The subjects were followed-up through quantitative determination of circulating SARS-CoV-2 S1/S2 IgG and neutralizing antibodies in the 6-month period after clinical and laboratory recovery. A group of 143 healthcare workers with no underlying chronic pathologies or renal diseases recovered from COVID was also evaluated. In both dialysis and transplanted patients, antibody titers reached a zenith around the 3rd month, and then a decline occurred on average between the 270th and 300th day. Immunocompromised patients who lost antibodies around the 6th month were more common than non-renal subjects, although the difference was not significant (38.5% vs. 26.6%). Considering the decay of antibody levels below the positivity threshold (15 AU/mL) as “failure”, a progressive loss of immunisation was found in the overall population starting 6 months after recovery. A longer overall antibody persistence was observed in severe forms of COVID-19 (*p* = 0.0183), but within each group, given the small number of patients, the difference was not significant (dialysis: *p* = 0.0702; transplant: *p* = 0.1899). These data suggest that immunocompromised renal patients recovered from COVID-19 have weakened and heterogeneous humoral responses that tend to decay over time. Despite interindividual variability, an association emerged between antibody persistence and clinical severity, similar to the subjects with preserved immune function.

## 1. Introduction

The coronavirus disease 2019 (COVID-19) pandemic is having a significant impact on subjects with impaired renal function [1,2,3,4], on patients under dialysis treatment [5,6] and kidney transplant recipients [7], with significantly higher infection rates compared to the general population [8,9,10]. The incidence of SARS-CoV-2 infection among patients with kidney failure remained of extreme clinical relevance even during the second pandemic wave, despite the preventive actions taken by all the nephrology centers [11]. Clinical outcomes confirmed the fragility of these patients with approximately 40% mortality in the hemodialysis population [12,13] and up to 30% in transplant recipients [14,15]. Obesity, diabetes, old age, and the presence of cardiovascular comorbidities represent negative prognostic factors [16,17,18,19]. The immune response has a central relevance for the course and severity of COVID-19 [20,21]. In this context, a better awareness should be achieved on the candidate factors able to modulate the immune system into a protective state or conversely trigger the particularly detrimental cytokine storm.

Over the months of the pandemic outbreak, the range of laboratory investigations available for diagnosis and monitoring of the infection has been progressively broadened. A growing attention has been focused on the role of acquired immunity and the persistence of anti-SARS-CoV-2 antibodies, with related benefits in terms of protection. At present, the gold standard to assess protective immunity against COVID-19 remains the detection of neutralizing antibodies.

However, the current available literature is especially focused on the general population. Antibody kinetics have been documented mainly in subjects with normal immune function. Patients with the severe form of the disease appear to develop higher peaks of neutralizing antibody titers compared to those with mild or asymptomatic infections, regardless of age [22]. Unfortunately, in spite of the several efforts to describe the medium- and long-term (i.e., >6 months) kinetics of anti-SARS-CoV-2 antibodies, data on the waning effect of the humoral response are not fully reliable, yet [23]. At present, the permanence of the state of immunity is not quantifiable with certainty. In addition, the puzzling matter of the durability of protection in immunocompromised patients is even less clear. We have previously reported a trend towards a delayed virus clearance in patients under dialysis treatment and renal transplant recipients. The presence of anti-SARS-CoV-2 antibodies, measured by qualitative and/or semi-quantitative tests, was detected a few days after recovery from the infection, and this positive serological response persisted during the first two months of observation at least [24]. However, in such a weak population, longer-term data about the effective achievement of immune protection and its steady durability are still lacking.

The aim of this work was to verify the ability to acquire and maintain protective antibodies over time after clinical and laboratory recovery from COVID-19 in patients under chronic dialysis and in kidney transplant recipients.

## 2. Results

### 2.1. Patients

The cohort analysed in this study consisted of 47 total patients, 26 under chronic hemodialysis and 21 renal transplant recipients. A group of 143 healthcare workers recovered from COVID-19 was also included. All the participants were followed-up through serological assessments in the 6-month period following laboratory and clinical recovery and prior to vaccination. The range of follow-up was 0.4–12.6 months (median: 4.6 months; IQR: 2.8–8.2 months) for dialysis patients, and 0.2–12.6 months (median: 2.7 months, IQR: 1.6–3.6 months) for transplant recipients. In the cohort of 47 renal patients, the median time for the first serological assessment (from clinical and laboratory recovery to the first antibody testing) was 38 days, and the interval between the two successive checks was 53 ± 19 days. As shown in Table 1, dialysis and transplant groups were similar in terms of their main demographic, clinical, biochemical and hematology features—with the exception of age, as the dialysis patients were older. At the various blood tests following COVID-19 diagnosis, there were no significant variations over time for hemoglobin, white blood cells, platelet count, CRP, LDH, or ALT. At inter-group comparisons, only CRP was found to be higher in dialysis patients compared to transplant recipients (*p* = 0.039).

Among the 26 hemodialysis patients, the severity of lung involvement due to COVID-19 was as follows: 9 patients were asymptomatic, 8 had mild symptoms with no radiology-detectable lesions, 8 experienced bilateral interstitial pneumonia and 1 had acute respiratory distress syndrome (ARDS) with need of hyperbaric oxygen therapy (HBOT). In the group of 21 renal transplant recipients, 2 were asymptomatic, 9 showed mild symptoms with no radiology-detectable lesions, 8 had bilateral interstitial pneumonia and 2 had ARDS with necessity of HBOT.

The time for viral clearance, expressed as the interval between the diagnosis of infection and recovery based on a negative nasopharyngeal swab was 27.4 ± 14.8 days in dialysis patients, and 30.1 ± 21.3 days in transplant recipients.

In the dialysis group, 13 out 26 patients required hospitalization due to COVID-19 with a median length of hospital stay of 12.5 days. Among transplant recipients, 10 out 21 patients were hospitalized with a median length of hospitalization of 9.5 days.

In general, the average rate of hospitalization was higher during the first wave compared to the second wave of the pandemic (65% vs. 39%, respectively), as in the latter period, hospitalisations were limited to symptomatic (mild/severe) cases. Therapy regimens for COVID-19 were chosen according to the acquired clinical knowledge and experience of the center. As a general rule, no specific antiviral therapy was given to asymptomatic patients. Conversely, in symptomatic patients, steroid therapy was introduced or enhanced in association with unfractionated heparin or low molecular weight heparin. In transplanted patients, immunosuppressive therapy was reduced with withdrawal of the third immunosuppressant, when present. Antiviral therapy with Remdesivir was administrated to 10 out 21 (47.6%) transplant recipients. Oxygen therapy was given according to the individual’s clinical needs with respect to oxygen saturation.

### 2.2. SARS-CoV-2 S1/S2 IgG

To evaluate the course of antibody titers over time, the data of the quantitative SARS-CoV-2 S1/S2 IgG determinations were grouped according to the period after clinical and laboratory recovery: T1 (0–30 days), T2 (31–90 days), T3 (91–180 days), and T4 (181–300 days). Table 2 reports the quantitative SARS-CoV-2 S1/S2 IgG titers at the different time points in dialysis patients and renal transplant recipients.

**Figure 1:**
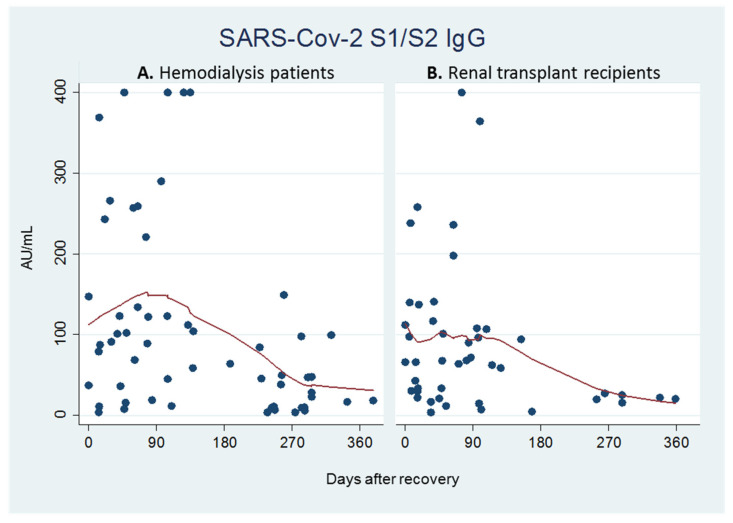
Quantitative antibody titers over time starting from day 0 (time of clinical and laboratory recovery) to the last observation available in hemodialysis patients (**A**) and renal transplant recipients (**B**).

The kinetics of the quantitative antibody titers from the time of clinical and laboratory recovery until the last serology assessment available are plotted in Figure 1A (hemodialysis patients) and 1B (transplant recipients). In both groups, the lowest smoothing curve method highlighted a trend towards a zenith around the 3rd month and then a decline until the last observation (on average between the 270th and 300th day).

To better clarify the time course data of each subject, individual plots of the 26 hemodialysis patients and 21 kidney transplant recipients are provided in Supplementary Figures S1 and S2, respectively.

**Figure 2:**
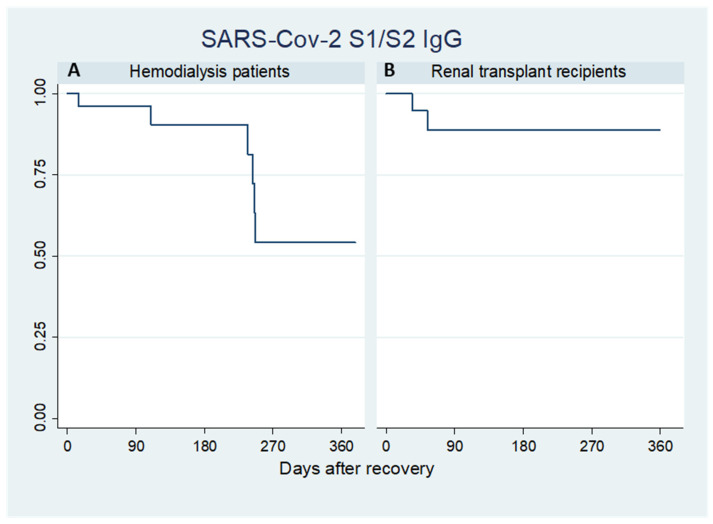
Kaplan–Meier plots of survival of SARS-CoV-2 S1/S2 IgG in hemodialysis patients (**A**) and renal transplant recipients (**B**).

Survival analysis by the Kaplan–Meier method was used to evaluate the persistence of SARS-CoV-2 S1/S2 IgG in hemodialysis patients (Figure 2A) and renal transplant recipients (Figure 2B). In general, a persistence of humoral response was observed for the first 6 months after recovery, with an antibody survival rate of 90.3% (95% CI: 65.9–97.6%) for dialysis patients and of 88.8% (95% CI: 62.1–97.1%) for transplant recipients, followed by a progressive antibody loss. However, it should be specified that the last portion of Kaplan–Meier survival curve, in particular after the 9th month in the transplant group, is based on a too-limited number of observations to be generalized.

A group of 143 healthcare workers (41 M, 102 F; age 44.3 ± 9.9 years) with no underlying chronic pathologies or renal diseases recovered from COVID was also evaluated with a serology assessment around 6 months after negativization. In general, considering the whole cohort of immunocompromised patients, a greater tendency towards a loss of antibodies below the positivity threshold of 15 AU/mL around the 6th month was noticed in comparison with the subjects with normal renal function, although the difference did not meet statistical significance (38.5% vs. 26.6%).

### 2.3. Neutralizing Antibodies

**Figure 3:**
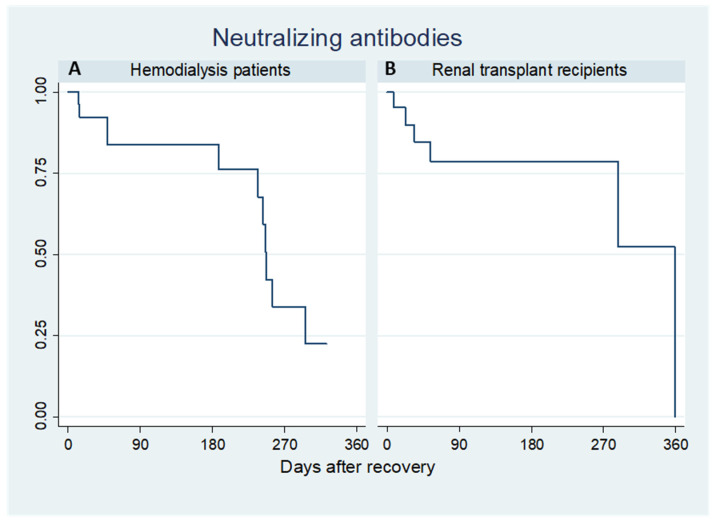
Kaplan–Meier plots of survival of neutralizing antibodies in hemodialysis patients (**A**) and renal transplant recipients (**B**).

The positive samples at quantitative serological assessment were retested by PRNT. Kaplan–Meier analysis for neutralizing antibodies revealed that in dialysis patients (Figure 3A) survival was 92.1% (95% CI: 0.721–0.980) at day 30, 83.8% (95% CI: 0.623–0.936) from day 90 to day 180, then there was a drop to 33.8% (95% CI: 0.109–0.588) at day 270. In the transplant group (Figure 3B), there was a slight decline of neutralizing antibody survival from 89.9% (95% CI: 0.653–0.974) at day 30 to 78.6% (95% CI: 0.522–0.915) from day 180 until day 270.

### 2.4. Factors Associated to Antibody Responses

**Figure 4:**
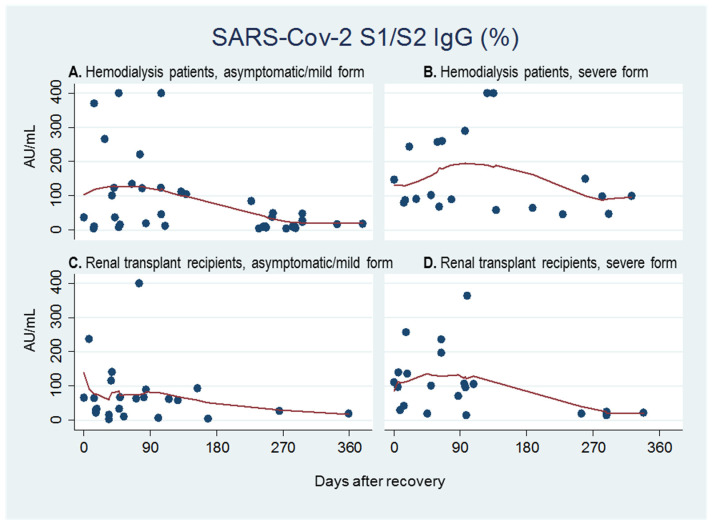
Quantitative antibody titers over time starting from day 0 (time of clinical and laboratory recovery) to the last observation available in hemodialysis patients with the asymptomatic/mild form (**A**) or the severe form of COVID-19 (**B**), and renal transplant recipients with the asymptomatic/mild form (**C**) or the severe form of COVID-19 (**D**).

Considering the severity of COVID-related lung involvement, the analysis was conducted on dichotomized variables: asymptomatic/mild symptoms versus severe, which included bilateral interstitial pneumonia and acute ARDS requiring HBOT. In the overall analysis of the whole cohort for SARS-CoV-2 S1/S2 IgG titers, those patients who experienced the severe form of the disease had also higher circulating antibody concentrations (*p* = 0.0460). This trend was statistically significant in the period between day 180 and day 300 (median: 117.5 AU/mL; range: 11.5–400 AU/mL; IQR: 58.8–400.0 AU/mL) in the dialysis group (Kruskal–Wallis test, *p* = 0.0095), and the period between day 90 and day 180 (median: 69.9 AU/mL; range: 3.8–400.0 AU/mL; IQR: 27.2–129.1 AU/mL) in the kidney transplant group (Kruskal–Wallis test, *p* = 0.0472; Figure 4).

The analysis of the factors associated with the persistence or loss of SARS-CoV-2 S1/S2 IgG and of neutralizing antibodies through the log-rank test revealed no significant impact of age, gender, dialysis vintage (for dialysis group), time after transplant and allograft function (for transplant recipients), initial viral load, clinical onset, therapy, or blood parameters. In the overall population of immunodepressed patients, a longer antibody persistence was observed in the more severe forms of the disease compared with the mild or asymptomatic forms (*p* = 0.0183). However, considering the individual groups of dialysis and transplanted patients, given the small number of cases, this difference did not meet statistical significance (*p* = 0.0702 in dialysis group and *p* = 0.1899 in transplant group).

**Figure 5:**
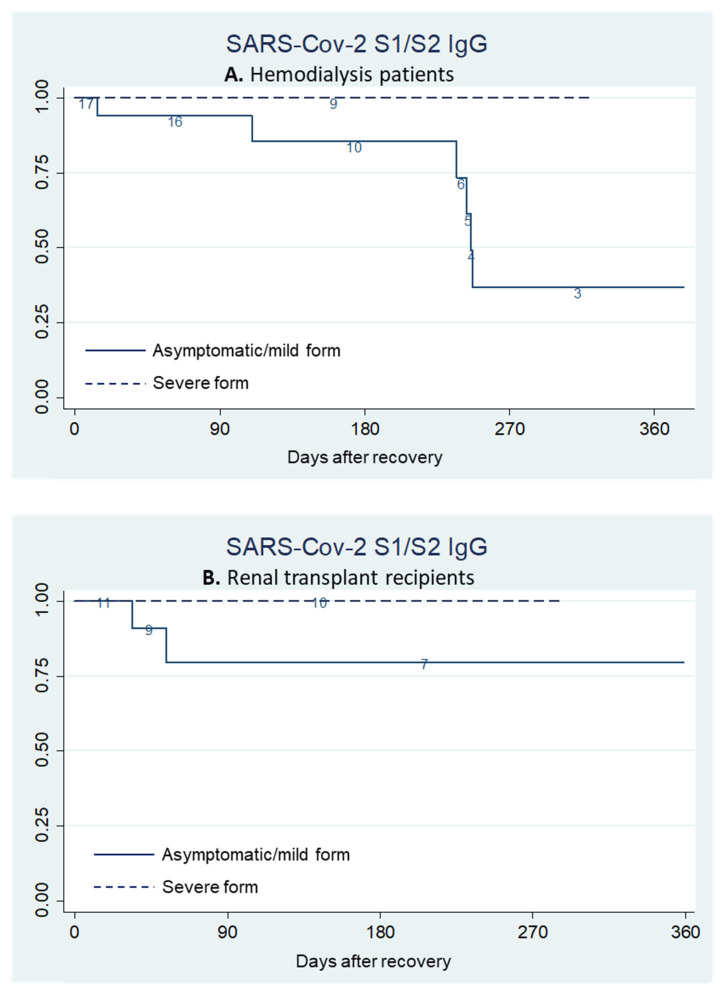
Kaplan–Meier plots of survival of SARS-CoV-2 S1/S2 IgG in COVID-19 asymptomatic/mild form (solid line) and in severe form (dashed line) in hemodialysis patients (**A**) and renal transplant recipients (**B**).

The Kaplan–Meier method, used to evaluate the trends in SARS-CoV-2 S1/S2 IgG survival according to the severity of the disease, confirmed that in both dialysis (Figure 5A) and transplanted patients (Figure 5B), those who experienced the severe form of COVID-19 had higher initial titers and a longer persistence of circulating antibodies compared to those with no or mild symptoms. Conversely, no significant correlations emerged between the severity of the disease and the trends of neutralizing antibodies.

## 3. Discussion

The dramatic impact of SARS-CoV-2 infection in terms of incidence and mortality among nephropathic patients was confirmed during the second pandemic wave in Italy [25]. It is well known that dialysis patients and transplant recipients are burdened by significant comorbidities and have an altered capacity of both humoral and cellular immune responses [24,26,27,28]. The pathogenetic link between uremia and immunodepression feasibly lies in the detrimental effects of the uremic milieu itself and the related disorders on immunocompetent cells, thought different molecular and cellular pathways. Disturbances of the innate immune system result in altered regulation of pattern-recognition receptors, monocyte and neutrophil hyporeactivity, and insufficient complement response. Besides this, acquired immunity is also compromised by the uremic state, via impaired activation of T-lymphocytes with concurrent increased Th1/Th2 ratio, decreased B-cell count, and altered function of APCs [29]. Even, before the COVID-19 pandemic, dialysis patients have been reported to have a delayed ability to eradicate viral infections for viruses other than SARS-CoV-2—namely, HBV, HCV, and HIV [30]. Previous data from our group have confirmed a longer viral clearance for SARS-CoV-2 infection as well [24]. In renal transplant recipients, although the negative effects of the uremic state are largely reverted by the restored kidney function, immune responses are compromised by the necessity of life-long anti-rejection therapy.

Our data are consistent with very recent evidence, and indications from several health and pharmacovigilance authorities have indicated dialysis patients and organ transplant recipients as a weak category with priority to receive a third vaccine booster dose to produce an adequate humoral response [31].

This complex scenario is often accompanied by a subclinical inflammatory status, which is likely to adversely affect outcomes, although at present it is not possible at present to provide precise individual prognostic indications [32]. It is also conceivable that genetic factors might play an important role in the inflammatory and immune response to COVID-19 [33,34].

This experience highlights some changes in the management of the infection in such fragile patients between the first and second pandemic waves. In particular, compared with our previous observation [24], we now found a lower rate of hospitalisation, currently restricted only to those patients with clinical symptoms of medium or high severity. Moreover, a more targeted approach in terms of drug interventions has been perceived during the last few months. Both of these aspects result from the clinical and management experience acquired in the first half of 2020. The post-infection serological data available up until now in immunocompromised patients are mainly qualitative or semi-quantitative. The possibility of quantifying and analyzing the antibody response and persistence represents a first landmark of great interest, especially in view of the intense vaccination campaigns with organizational and management rebounds in a population at high infection risk. Recent reports from the COVID-19 Survey by the Italian Society of Nephrology confirmed the elevated incidence of infection and the high death rates in patients with a long previous clinical history of renal failure [35].

Emerging data indicate that in the general population, seropositive COVID-19 recovered patients are protected against reinfection by approximately 90% [36], but the duration of protective immunity remains to be established. Unfortunately, there is some evidence to indicate that this immune response is likely to fade within few months [37], and besides, the emerging new variants of SARS-CoV-2 virus might be resistant to specific antibodies developed against older strains [38]. The ongoing studies on the factors able to influence the achievement of adequate protection against SARS-CoV-2 infection and its persistence over time represent a hot topic of current research, with the goal of tailoring vaccination and immunotherapy protocols. Patients with impaired renal function commonly show a poorer humoral immune response with a less durable neutralizing function against infections [39,40]. To confirm this view, we evaluated a group of healthcare workers with no comorbidities or renal disease at six months following recovery from COVID-19. In general, we noted that dialysis and transplanted patients who experienced antibody loss below the positive threshold of 15 AU/mL around the 6th month after clinical and laboratory recovery were more common than subjects with normal renal function, in line with recently published data [41]. Recently, Muir and colleagues investigated antibody responses to SARS-CoV-2 and neutralizing activity in 164 nephropathic patients (pre-dialysis, peritoneal dialysis, and hemodialysis) awaiting renal transplant. The authors found a higher and long-lasting seroprevalence (anti-S1 and/or anti-N IgG) in patients on hemodialysis, although a significant drop in S1, N and nAb titers was observed after the third month [42].

Our findings in the overall cohort of immunodepressed subjects seem to confirm such heterogeneity in humoral immune responses that tends to decline over time. A longer antibody persistence and higher initial SARS-CoV-2 S1/S2 IgG titers are associated with the severity of the disease, in agreement with evidence from non-renal patients [43]. In our experience, some patients had no detectable antibodies (SARS-CoV-2 S1/S2 IgG < 15 AU/mL) from the first quantitative test, thus the downward steps in the first part of the Kaplan–Meier survival curve should not be interpreted as an early antibody loss, but more feasibly as a lack of development. This hypothesis was confirmed by the fact that those patients with no antibody detectable titers at the first serological assessment maintained their negativity at successive checks. Of note, those subjects that tested negative since the beginning of our quantitative serological monitoring were asymptomatic or paucisymptomatic and this might represent a further possible explanation for the missing antibody response. Taken together, these findings seem to confirm the fragility of these patient populations, which appear more prone to develop a weakened immune response and to rapidly lose protection against COVID-19.

We found that the drop in both SARS-CoV-2 S1/S2 IgG titers and neutralizing antibodies was faster and more pronounced in dialysis group, whereas a milder decline was observed in transplant recipients. Particular attention should be also be given to the systematic and repeated use of diagnostic tests for the early diagnosis of COVID-19 (in particular rapid antigen-testing and molecular testing) in weak patients at higher infective risk. Currently, the strategy to eliminate COVID-19 is based on mass vaccination campaigns. Thus, a better awareness of the immune responses to SARS-CoV-2 infection in immunodepressed populations is also important to optimize vaccination strategies in such weak patients [44].

This study has some limitations: we analysed a relatively small group with some heterogeneity, particularly in the timing of the analysis with respect to the period of infection. No data on antibody trends over the course of infection are currently available, since our serological tests refer to the period after clinical and laboratory recovery. Moreover, this study is focused only on humoral immune response aspects; the cell response was not investigated. Concerning the viral strain, we did not analyze gene sequence, but in view of the period of our observations, the infections are feasibly not due to the more recent variants.

At the moment, there is no consensus about the cut-off value of antibodies able to confer effective protection; moreover, the consistency between the dynamics of S1/S2 IgG and neutralizing antibodies is not fully understood [45]. In the near future, further prospective studies on a larger number of cases are needed to assess the permanence (or lack thereof) of immune protection, also taking the contribution of cellular responses into account.

In spite of these weak points, the main attractiveness of our current results lies in the perspective design with a relatively long observation period, together with the practical implications for vaccination programmes that can also be extended to other types of fragile populations (e.g., cancer patients). In Italy, the vaccination campaign has included dialysis or transplanted patients in the category of extremely vulnerable subjects and has allowed them to be vaccinated with priority criteria. In subjects with impaired immune response, a better awareness of antibody kinetics over time might be helpful in optimization of vaccination programs that might require increased doses (similarly to hepatitis B vaccination) or more shots to achieve effective protection. Very recent evidence suggests the utility of a boost with a third vaccine dose for immunodepressed populations [31,46,47,48,49]. Preliminary data by Del Bello et al. on 396 solid organ transplant recipients reported a prevalence of anti-SARS-CoV-2 IgG of 1.3% before the first dose of the messenger RNA-based BNT162b2 vaccine (Pfizer-BioNTech), 5.1% before the second, 41.4% before the third, and 67.9% after 4 weeks from the third injection, with 105 out of 232 patients (45.25%) seronegative prior to the boost with the third dose who then turned positive [31]. Similar results have been described in kidney transplant recipients who had not responded to two initial doses of the mRNA-1273 vaccine (Moderna) and who then received a third booster dose. Among the analysed population, 49% were able to develop SARS-CoV-2 antibodies after the third injection, while the remaining 51% who remained negative were predominantly the ones under triple immunosuppression [49].

## 4. Materials and Methods

### 4.1. Study Design and Participants

This is a multicenter study observational study to evaluate the extent and persistence of humoral response to SARS-CoV-2 infection in dialysis patients and renal transplant recipients recovered from COVID-19 at the Nephrology and Dialysis Units of the local health authority of Romagna (Cesena, Forlì, Ravenna, and Rimini), an area with a total reference population of 1,200,000 inhabitants.

From December 2020 to May 2021, all the dialyzed or transplanted patients diagnosed with COVID-19 during 2020 who achieved clinical and laboratory recovery (documented by a negative molecular nasopharyngeal swab) were followed to monitor the level of acquired immunization for an average period of 6 months. A group consisting of 143 healthcare workers followed up in the 6-month period after laboratory and clinical recovery from COVID-19 but prior to vaccination was also included. The participants were infected during the first or second waves of the pandemic. Specifically, the study population consisted of 47 patients—26 of them under chronic dialysis treatment at the time of infection and 21 clinically stable renal transplant recipients.

The first serological assessment was carried out at least 15 days after recovery and the successive checks were scheduled every 2 (±0.5) months. The overall follow-up period for each immunosuppressed patient started at the time of infection and ended in case of one of the following situations: (i) last serological assessment (second or third SARS-CoV-2 antibody testing); (ii) negative serological result; or (iii) vaccination.

For all the patients, the following general, clinical and infection-related parameters were collected: age, gender, severity of lung involvement, COVID-related symptomatology, drug regimen for COVID-19, time for viral clearance (interval between the first positive swab and the first negative swab), dialysis vintage (for dialysis patients), time after transplant, immunosuppressive therapy, and graft function (for transplant recipients).

The study was approved by the Institutional Ethics Committee (Comitato Etico della Romagna, CEROM) on 11th December 2020 with code INCoV19ID, and conformed to the principles of the Declaration of Helsinki. Written informed consent was provided by all the patients and the data were fully anonymized.

### 4.2. Laboratory Assays

Dialysis and transplanted patients were assayed for the following hematological parameters using standard laboratory methods: hemoglobin, white blood cell count, platelet count, serum creatinine, C-reactive protein (CRP), lactate dehydrogenase (LDH), serum protein electrophoresis, and alanine aminotransferase (ALT).

**Figure 6:**
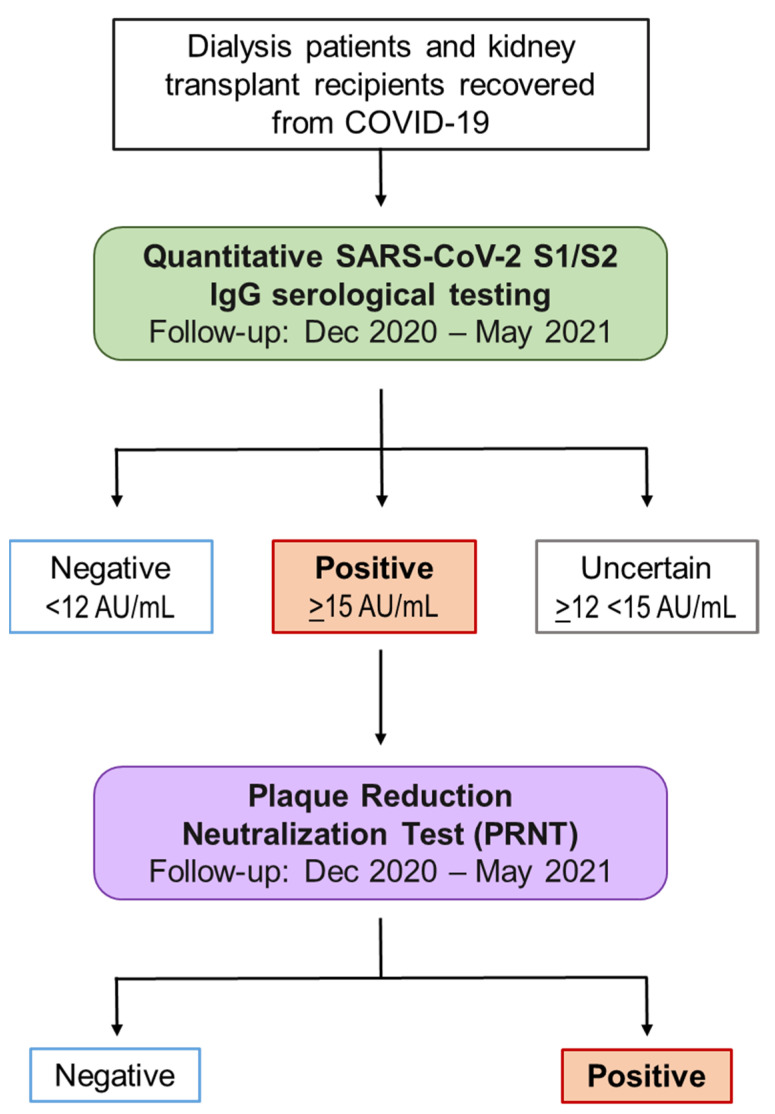
Algorithm of antibody testing. From December 2020 to May 2021, dialysis patients and transplant recipients recovered from COVID-19 during 2020 followed up to monitor the level of acquired immunization for a period of at least 6 months. The samples that resulted positive at quantitative testing (antibody titer ≥ 15 AU/mL) were retested with the plaque reduction neutralization test (PRNT) to assess neutralizing antibodies.

Antibody kinetics were prospectively monitored through repeated quantitative determinations of anti-SARS CoV-2 Spike protein IgG and the titer of neutralizing antibodies. The algorithm of antibody testing used to evaluate the immunodepressed population is illustrated in Figure 6.

Antibody serum levels were measured using an indirect chemiluminescence immunoassay (CLIA) for the quantitative determination of anti-S1 and anti-S2 IgG antibodies to SARS-CoV-2 (LIAISON^®^ SARS-CoV-2 S1/S2 IgG, DiaSorin, Saluggia, Italy), fully automated on the LIAISON^®^ XL Analyzer. The coronavirus spike (S) glycoprotein is a trimeric transmembrane protein protruding from the viral surface that mediates the virus entry into host cell. Each spike glycoprotein monomer consists of two functional subunits: S1 for binding to receptor ACE2 of the host cell, and S2 for the fusion of viral and host cell membranes [50]. The immunoassay used here is based on the detection of specific antibodies to recombinant S1 and S2 antigens. The concentrations of SARS-CoV-2 S1/S2 IgG are expressed as arbitrary units/mL (AU/mL). The results were considered as positive if ≥15 AU/mL, uncertain if <15 AU/mL and ≥12 AU/mL, and negative if <12 AU/mL.

In order to assess the neutralization activity of SARS-CoV-2 spike antibodies, all the positive samples were submitted to the plaque reduction neutralization test (PRNT), referred to as the gold standard technique to determine the highest serum dilutions that can neutralize the cytopathic effect (plaque formation) of the virus on cell cultures. The viral suspension VR PV10734 was supplied by the Virology Laboratory of Policlinico San Matteo in Pavia (Italy). This strain was isolated before the spread of current variants and corresponds to lineage B.1/D614G (GISAID code EPI_ISL_1908157). The viral strain was sequenced using Clean Plex SARS-CoV-2 Flex (Paragon Genomics Inc., Hayward, CA, USA) and Illumina MiSeq (Illumina Inc., San Diego, CA, USA). Viral titers, expressed as TCID50/mL (median tissue culture infectious dose), were calculated according to Reed and Muench [50,51] and Sperman–Karber methods, based on eight replicates for dilution [52,53,54]. The Vero E6 cell line was supplied by the National Institute for Infectious Diseases, IRCCS, Lazzaro Spallanzani (Rome, Italy). The test was performed as previously described [55]. Briefly, the cells were resuspended in a liquid culture medium and transferred into a 96-well cell culture plate containing a sera-virus mixture. After 72 h following infection, the cells were fixed and stained. The dye selectively binds the living cells fixed to the well, thus it is able to discriminate living from the dead cells. The first serum dilution considered was 1:10 (10 titer) and the highest serum dilution able to neutralize 90% (IC90) of the cytopathic effect is reported as the neutralizing titer.

### 4.3. Statistical Analysis

Continuous variables are presented as mean ± standard deviation (SD) for normally distributed data, or as median with minimum, maximum, interquartile range for skewed distributions, and categorical variables as absolute numbers with percentages, as appropriate. The differences between subgroups of antibody titres were tested with the non-parametric Kruskal–Wallis rank sum test. The persistence of antibody levels above the threshold value was studied using survival analysis with Kaplan–Meier method. The variable “failure” was defined as the loss of antibodies and the variable “time” was the interval between the date of the last sampling and the date of clinical and laboratory recovery. The possible associations between antibody survival and patient characteristics were analyzed through the log-rank test for categorical variables and the univariate Cox model for continuous variables. A *p* value < 0.05 was considered significant. Data analysis was performed using the statistical package Stata (version 14.2, Stata Corporation).

## 5. Conclusions

In conclusion, our investigation reveals an impaired and heterogeneous serological protection in immunocompromised renal patients recovered from COVID-19 that tends to fade over time. Even in the context of a wide interindividual variability, we highlighted a relationship between the extent of humoral responses (peak levels, antibody persistence) and the clinical severity of infection, similar to subjects with preserved immune function. A clearer vision might be reached after an in-depth prospective analysis of a large number of cases.

## Figures and Tables

**Table 1:** Main general, clinical, biochemical and hematology features of the 47 immunodepressed patients who recovered from COVID 19; 26 patients under chronic dialysis treatment and 21 renal transplant recipients. Continuous variables are presented as means ± standard deviation, and categorical variables as absolute numbers and percentage in brackets. * *p* < 0.05.

	TOTAL (n = 47)	Hemodialysis Patients (n = 26)	Renal Transplant Recipients (n = 21)
Age, years	60.1 ± 15.2	65.5 ± 15.0 *	53.7 ± 13.1 *
Gender, M (%)	36 (76.6%)	20 (76.9%)	16 (76.2%)
Dialysis vintage, months	/	33.1 ± 27.9	/
Transplant age, months	/	/	75.3 ± 59.32
sCreat at baseline, mg/dL	/	/	1.5 ± 0.7
sCreat at recovery, mg/dL	/	/	1.6 ± 0.6
Hemoglobin, mg/dL	11.9 ± 1.7	11.6 ± 1.5	12.3 ± 1.8
WBC, 10^3^ cells/µL	7.1 ± 2.4	7.4 ± 2.6	6.7 ± 2.0
Platelet count, 10^3^ cells/µL	211 ± 70	206 ± 67	218 ± 75
CRP, mg/dL	4.7 ± 7.2	6.3 ± 8.7 *	2.4 ± 2.7 *
LDH, mg/dL	210 ± 53	211 ± 56	209 ± 50
ALT, mg/dL	14.7 ± 10.4	12.4 ± 9.9	18.0 ± 10.3
Degree of respiratory distress			
None/mild	28 (59.6%)	17 (65.4%)	11 (52.4%)
Severe	19 (40.4%)	9 (34.6%)	10 (47.6%)

sCreat, serum creatinine; WBC, white blood cell count; CRP, C-reactive protein; LDH, lactate dehydrogenase; ALT, alanine aminotransferase.

**Table 2:** SARS-CoV-2 S1/S2 IgG titers expressed as AU/mL of the positive serum samples at T1 (0–30 days), T2 (31–90 days), T3 (91–180 days), and T4 (181–300 days) after clinical and laboratory recovery in dialysis patients and renal transplant recipients (with the related number of available tests in brackets). Data are presented as median, range, and interquartile range (IQR).

		Median, AU/mL	Range, AU/mL	IQR, AU/mL
**Hemodialysis** **patients**	T1: 0–30 days (n = 13)	89.2	3.8–369.0	37.5–243.0
	T2: 31–90 days (n = 14)	102.0	7.9–400.0	36.4–221.0
	T3: 91–180 days (n = 9)	117.5	11.5–400.0	58.8–400.0
	T4: 181–300 days (n = 12)	28.5	3.8–149.2	9.3–50.1
**Renal transplant recipients**	T1: 0–30 days (n = 17)	66.2	22.2–258.3	33.6–137.3
	T2: 31–90 days (n = 17)	69.9	3.8–400.0	27.2–129.1
	T3: 91–180 days (n = 5)	78.2	4.9–364.0	15.2–107.1
	T4: 181–300 days (n = 2)	22.7	15.7–27.4	18.0–26.3

## Data Availability

The data presented in this study are available on request from the corresponding author.

## Supplementary Materials

The following are available online at www.mdpi.com/article/10.3390/pathogens10101289/s1, Figure S1: Single course of quantitative antibody titers over time starting from day 0 (time of clinical and laboratory recovery) to the last observation available in each of the 26 hemodialysis patients; Figure S2: Single course of quantitative antibody titers over time starting from day 0 (time of clinical and laboratory recovery) to the last observation available in each of the 21 renal transplant recipients.
