# Supplementary material for: Persistence of Antibody Responses to the SARS-CoV-2 in Dialysis Patients and Renal Transplant Recipients Recovered from COVID-19
Source: Pathogens. 2021 Oct 6;10(10):1289. doi: 10.3390/pathogens10101289 (PMC8541005; doi:10.3390/pathogens10101289)
Supplement: Supplementary file 1 [file pathogens-10-01289-s001.zip › pathogens-1364715-supplementary.pdf]

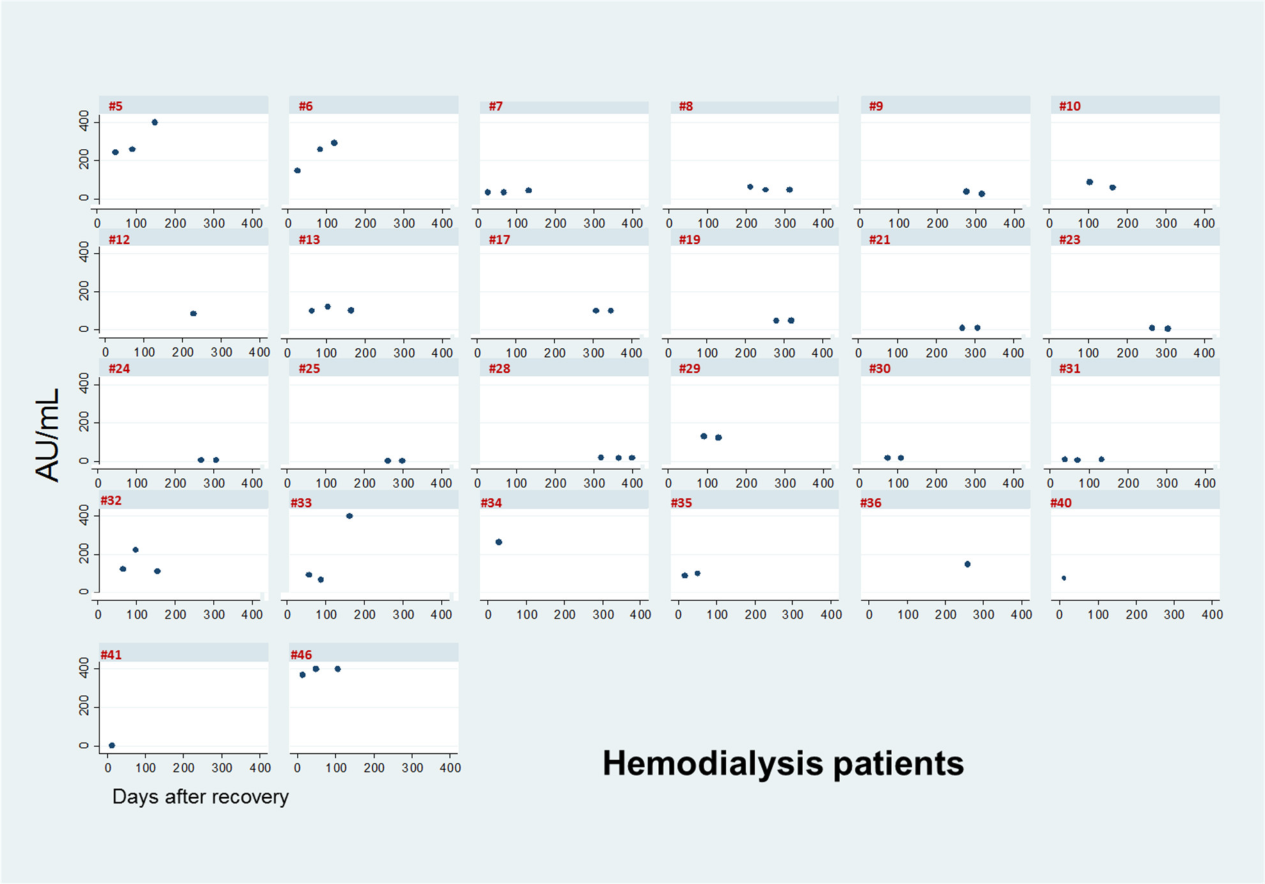

Figure 1S. Single course of quantitative antibody titers over time starting from day 0 (time of clinical and laboratory recovery) to the last observation available in each of the 26 hemodialysis patients.

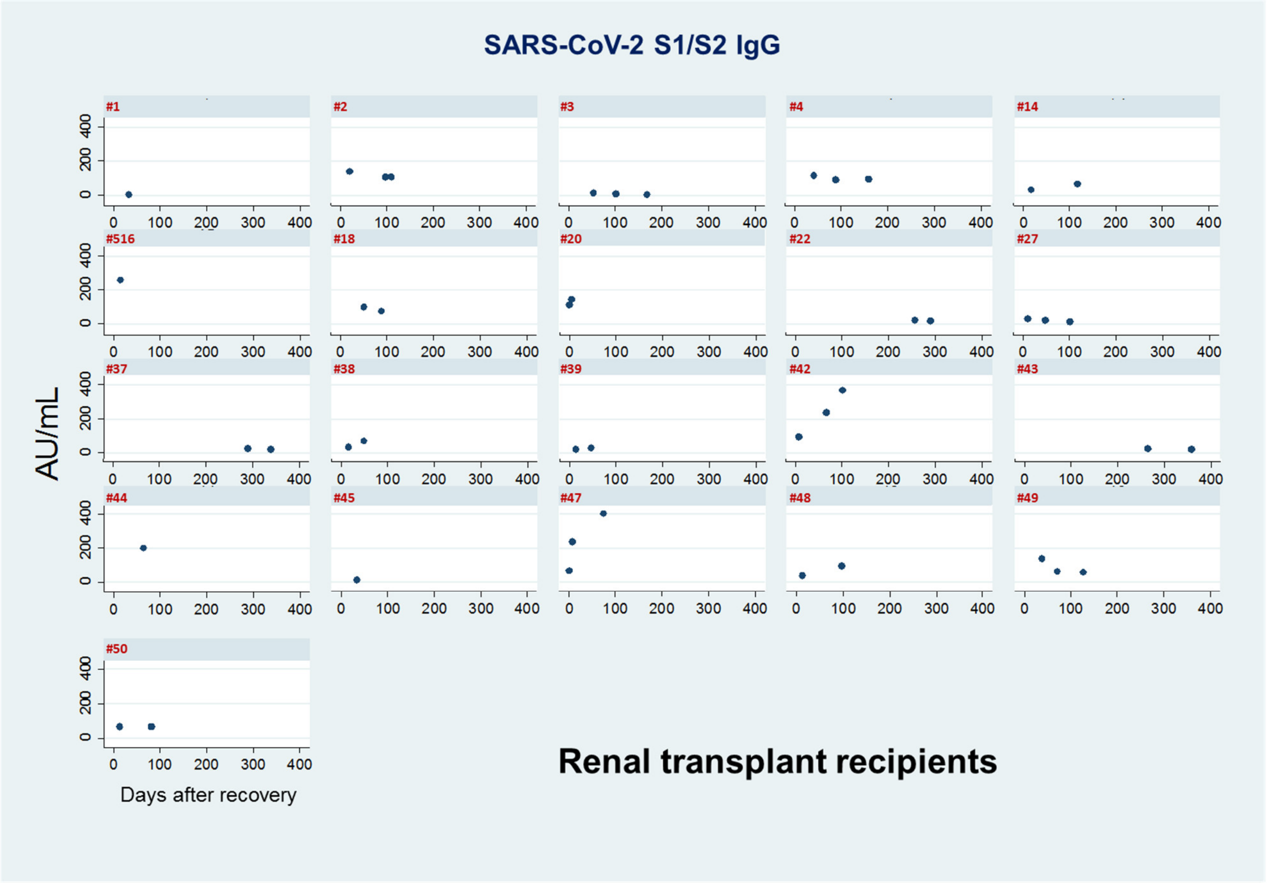

Figure 2S. Single course of quantitative antibody titers over time starting from day 0 (time of clinical and laboratory recovery) to the last observation available in each of the 21 renal transplant recipients.
